# High-precision neural stimulation by a highly efficient candle soot fiber optoacoustic emitter

**DOI:** 10.3389/fnins.2022.1005810

**Published:** 2022-11-03

**Authors:** Guo Chen, Linli Shi, Lu Lan, Runyu Wang, Yueming Li, Zhiyi Du, Mackenzie Hyman, Ji-Xin Cheng, Chen Yang

**Affiliations:** ^1^Department of Electrical and Computer Engineering, Boston University, Boston, MA, United States; ^2^Photonics Center, Boston University, Boston, MA, United States; ^3^Department of Chemistry, Boston University, Boston, MA, United States; ^4^Department of Mechanical Engineering, Boston University, Boston, MA, United States; ^5^Department of Biomedical Engineering, Boston University, Boston, MA, United States

**Keywords:** neural modulation, neural stimulation, optoacoustic stimulation, optoacoustic, photoacoustic, fiber, optoacoustic conversion efficiency

## Abstract

Highly precise neuromodulation with a high efficacy poses great importance in neuroscience. Here we developed a candle soot fiber optoacoustic emitter (CSFOE), capable of generating a high pressure of over 10 MPa with a central frequency of 12.8 MHz, enabling highly efficient neuromodulation *in vitro*. The design of the fiber optoacoustic emitter, including the choice of the material and the thickness of the layered structure, was optimized in both simulations and experiments. The optoacoustic conversion efficiency of the optimized CSFOE was found to be 10 times higher than the other carbon-based fiber optoacoustic emitters. Driven by a single laser, the CSFOE can perform dual-site optoacoustic activation of neurons, confirmed by calcium (Ca^2+^) imaging. Our work opens potential avenues for more complex and programmed control in neural circuits using a simple design for multisite neuromodulation *in vivo*.

## Introduction

Highly precise neural modulation is of great importance in neuroscience, as firing of specific neuronal populations in the brain could alter the behavior of animals and serve as a novel tool for studying neural pathways in disease and health. Sophisticated control of neuronal circuits and brain functions requires stimulating multiple functional regions at high spatial resolution. For example, a previous study by Li et al. (2019) used two ultrasound transducers to stimulate primary somatosensory cortex barrel field (S1BF) of a free moving mouse and successfully controlled the head turning direction of the mouse by applying stimuli at different positions. Among the current neuromodulation platforms, electrical neural stimulation has been proven to be efficient and allows for deep brain stimulation, while it provides a limited spatial resolution of millimeters *in vivo*, due to electric current spread (Boon et al., 2007). Optogenetics neural stimulation with single neuron resolution has been shown as a powerful tool in fundamental studies, but the requirement of viral infection makes it challenging to apply to human brains (Boyden et al., 2005). Transcranial magnetic stimulation (TMS) and transcranial direct current stimulation (tDCS) are capable of non-invasive transcranial neuromodulation, while suffering from the resolution at the centimeter level (Rosa and Lisanby, 2012; Davidson et al., 2020). Infrared neuron stimulation (INS) takes advantage of the near-infrared absorption of water to generate heat for neural stimulation. However, the thermal toxicity and potential tissue damage is a concern in real clinical scenarios (Cayce et al., 2014; Zhu et al., 2022). Low frequency focused ultrasound is an emerging non-invasive modality with centimeter-scale penetration depth in tissue (Beisteiner et al., 2020; Bobola et al., 2020; Brinker et al., 2020). It has a spatial resolution limited by the diffraction of acoustic wave, therefore it is challenging for low-frequency (< 1 MHz) ultrasound to reach submillimeter spatial resolution. New technologies and methods are still being sought for precise and non-genetic neural stimulation.

Recently, our team developed a miniaturized fiber-optoacoustic converter (FOC) converting pulsed laser into ultrasound (Jiang et al., 2020). FOC succeeded in spatially confined neural stimulation of mouse brain and modulation of motor activities *in vivo*. It was found that, for successful FOC based optoacoustic neural stimulation, a pressure of around 0.5 MPa is needed (Jiang et al., 2020). Typical FOC generate a pressure of 0.48 MPa upon laser pulse energy of 14.5 μJ, with an estimated photoacoustic conversion efficiency of 1,374 Pa m^2^/J. Considering the typical energy and repetition rate of nanosecond lasers, the low conversion efficiency of FOC limits its application in multi-site stimulation. Thus, new fiber optoacoustic emitters with a higher conversion efficiency are needed to enable multisite optoacoustic neuromodulation.

According to a simplified model of optoacoustic generation, the output optoacoustic pressure is related to the laser fluence (*F*), the absorption coefficient (α) and the thermal expansion coefficient (β) (Xu and Wang, 2006). The pressure generated can be calculated following the equation below:


P=Γ (β)αF

where the Grüneisen parameter (Γ) is a function of the thermal expansion coefficient β. To improve the optoacoustic conversion efficiency, materials with greater light absorption and larger thermal expansion coefficients are preferred. Previously, many materials have been studied for efficient optoacoustic conversion, including metal, carbon material, etc. Metals, in the form of gold nanoparticles (Wu et al., 2011, 2012, 2013; Tian et al., 2013; Zou et al., 2014) and Cr and Ti films (Lee and Guo, 2017) were used due to their high absorption coefficient. However, the high light reflection by metal films and scattering of metal nanoparticles limit the energy conversion efficiency. Different carbon materials have also been studied, including carbon nanoparticle (CNP) (Biagi et al., 2001), carbon nanotube (CNT) (Won Baac et al., 2010; Colchester et al., 2014; Baac et al., 2015; Alles et al., 2016; Noimark et al., 2016; Moon et al., 2017; Poduval et al., 2017; Shi et al., 2020; Thompson et al., 2022), graphite (Jiang et al., 2020) and candle soot films (CS) (Chang et al., 2015, 2018; Huang et al., 2016). Among these, candle soot stands out for its high light absorption coefficient, low interfacial thermal resistance, and easy fabrication process. Direct comparison of optoacoustic conversion efficiency among CS, CNT, and CNP showed CS can generate a pressure six times higher than that generated by the other two materials (Chang et al., 2018). The CS layer deposited onto polydimethylsiloxane (PDMS), a material with high thermal expansion coefficient (Wolf et al., 2018), forms a diffused mixture—an excellent choice for highly efficient optoacoustic generation.

In this work, we developed a candle soot-based fiber optoacoustic emitter (CSFOE) for the first time. COMSOL simulation was used to simulate the optoacoustic generation process of a CSFOE. We optimized the design of the CSFOE by identifying the optimal thickness of the CS layer through simulation. A CSFOE with a CS layer of an optimal ∼10 μm thickness was found to achieve the highest peak-to-peak pressure. Experimentally, we fabricated CSFOEs with controlled thicknesses of candle soot layers in the range of 1 μm to 60 μm. By comparing their optoacoustic performance, we confirmed that the optimal thickness of the CS layer is 10 μm, consistent with the simulation prediction. The maximum optoacoustic pressure reached ∼10 MPa, which is 9.6 times larger compared with that generated by FOC (Jiang et al., 2020; Shi et al., 2021). The application of CSFOE to non-genetic optoacoustic neural stimulation was demonstrated in GCaMP6f labeled neuron culture. Successful high precision activation of neurons confined in an area of 200 μm was verified by calcium imaging. Significantly, we demonstrated dual-site optoacoustic neural stimulation driven by a single laser utilizing the high optoacoustic conversion efficiency of CSFOE. The highly localized ultrasound field generated by each CSFOE allows the two stimulated sites to be sub-millimeter apart. Our work opens up potentials for complex and programmed control in neural circuits using a simple design for multisite neuromodulation.

## Materials and methods

### Simulation of the ultrasound field generated from candle soot-based fiber optoacoustic emitters

The ultrasound field generated by CSFOE was simulated by COMSOL Multiphysics 5.4. The CSFOE was modeled by a 2D axisymmetric model with a double-layer structure, including an absorber layer of CS and PDMS mixture and a pure PDMS layer. The backing material was set to be a fiber (SiO_2_). Radiative Beam in Absorbing Media module was used to simulate the absorption of the CSNP-PDMS mixture layer when being applied a nanosecond pulsed laser. The Heat Transfer and Solid Mechanics modules were used to model the thermal expansion caused by the photothermal effect. The Transient Pressure Acoustic module converted the thermal expansion of the absorber into the acoustic signal and simulated the propagation of ultrasound in the water medium. The absorption coefficient of the absorber layer used in the simulation was a measured value by our experiment, and all the other material parameters were set according to COMSOL’s material library database. The time step of the simulation was set to be 0.001 ns at the beginning to fully simulate the nanosecond laser pulse, and then to simply the calculation, the time step was increased to 5,000 ns after the first 10 ns of simulation. The mesh grid size was set to be a physics-controlled mesh in COMSOL and it can adjust its size automatically to a suitable value according to the simulation model.

### Fabrication of candle soot-based fiber optoacoustic emitters

**FIGURE 1:**
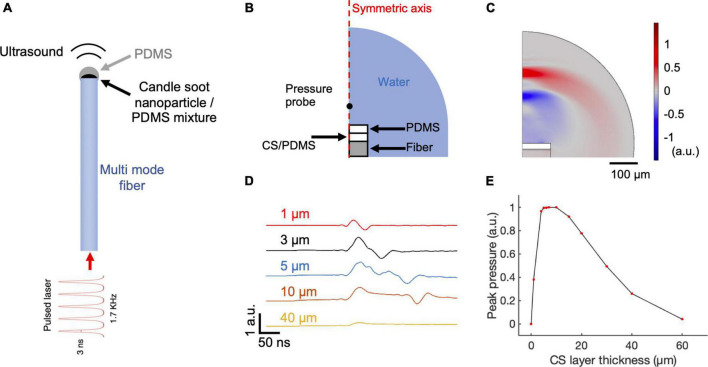
COMSOL simulation of CSFOE performance. **(A)** Schematic of CSFOE. **(B)** Illustration of the CSFOE model used in simulation. Not to scale. **(C)** Representative ultrasound waveform, simulated at *t* = 400 ns under an input of a 3 ns pulsed laser. **(D)** Acoustic waveforms simulated at different thicknesses of the CS layer. **(E)** Peak-to-peak acoustic pressure plotted as a function of candle soot layer thickness.

A schematic of the CSFOE is shown in Figure 1A. A flame from a paraffin wax candle served as the source of the candle soot. To fabricate the CSFOE, the tip of a polished multimode optical fiber with a 200 μm diameter (200EMT, Thorlabs) was placed into the center of the flame for 3–5 s. This step was repeated until the optical fiber was fully coated with flame synthesized candle soot. To prepare the PDMS, the silicone elastomer (Sylgard 184, Dow Corning Corporation, USA) was carefully dispensed into a container to minimize air entrapment. Then, the curing agent was added for a weight ratio of ten to one (silicone elastomer to curing agent). A nanoinjector deposited the PDMS onto the tip of the candle-soot coated fiber. The position of the fiber and the nanoinjector were both controlled by 3D manipulators for precise alignment, and the PDMS coating process was monitored under a lab-made microscope in real time. The coated fiber was stored overnight in a temperature-controlled environment (20°C) for 12 h, to allow the PDMS to cure and fully diffuse into the porous structure of the candle soot.

### Characterization of absorption ability of CS

The absorption of CS with different deposition thicknesses was measured with a photodiode (DET10A2, Thorlabs, USA, covering the detection range from 200 nm to 1,100 nm). Different thicknesses of CS were controlled by the deposition durations. The fiber was connected to a Q-switched 1,030-nm nanosecond laser (Bright Solution, Inc. Calgary Alberta, CA), and the transmission power was detected by the photodiode.

### Characterization of optoacoustic signals

**FIGURE 2:**
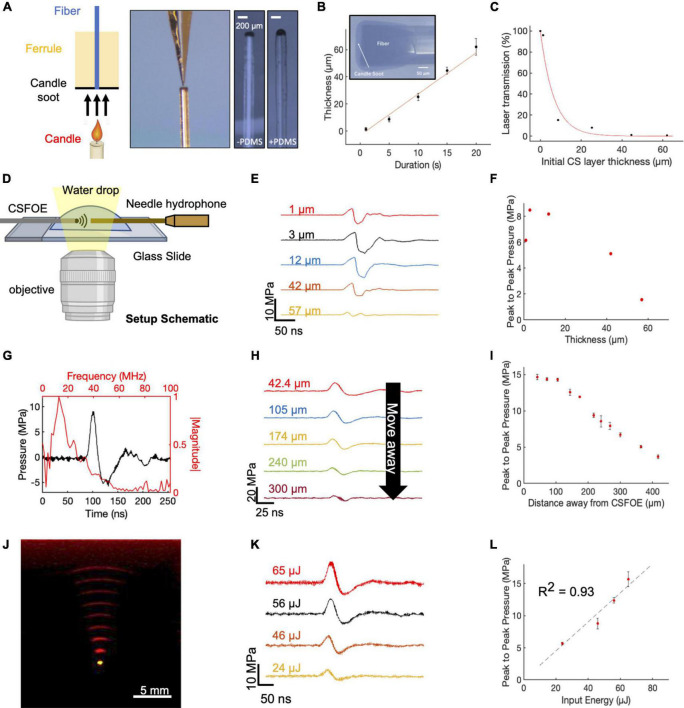
Fabrication and characterization of CSFOE. **(A)** Key steps of CSFOE fabrication. (Left) Candle soot deposition on an optical fiber tip. (Middle) PDMS coating on the surface of the CS layer using a nanoinjector. (Right) Images of samples after CS deposition and PDMS coating, respectively. Scale bars: 200 μm. **(B)** Thickness of the CS layer obtained as a function of deposition duration. Inset: representative image of a fiber coated with candle soot. **(C)** Transmission ratio plotted as a function of the thickness of CS layer. **(D)** Schematic of experimental configuration of photoacoustic signal measurement using a 40 μm needle hydrophone. Created with BioRender.com. **(E)** Acoustic signal of CSFOE as a function of the candle soot layer thickness detected by the hydrophone. Laser condition: 1,030 nm, 1.7 kHz repetition rate, 46 μJ per pulse. **(F)** Peak to peak pressure plotted as a function of the thickness of CS under the same laser condition as **(E)**. **(G)** Representative photoacoustic waveform (Black) detected by the hydrophone and its FFT frequency spectrum (Red). laser condition: 1,030 nm, 56 μJ pulse energy. **(H,I)** Acoustic signal and peak-to-peak pressure generated by CSFOE detected at different distances from the CSFOE tip. Each data point was an average of three trials. laser condition: 1,030 nm, 56 μJ pulse energy, distance between hydrophone and CSFOE: 70 μm **(J)** Photoacoustic signal propagation in the medium detected by a linear transducer array. Fiber tip (Yellow), PA waveform (red). **(K,L)** Photoacoustic waveforms and peak-to-peak pressures measured at different laser pulse input. Each data point was an average of three trials, distance between hydrophone and CSFOE: 70 μm.

The amplitude of the CSFOE-generated acoustic wave was measured using a needle hydrophone with a 40 μm core sensor (Precision Acoustics, UK). A digital oscilloscope (DSO6014A, Agilent Technologies, CA, USA) recorded the electrical signal from the hydrophone. A four-axis micro-manipulator (MC1000e controller with MX7600R motorized manipulator, Siskiyou Corporation, OR, USA), with a resolution of 0.2 μm, controlled the distance between the CSFOE tip and the hydrophone. For the measurement of optoacoustic signals (Figure 2F), CSFOE was aligned with the hydrophone using the micromanipulator until the signal amplitude reached its maximum. The distance between the hydrophone and the CSFOE was kept to be within the sub-millimeter range. The same alignment has been performed to all the CSFOE for Figure 2F.

For distance-dependent characterization of optoacoustic signal intensity in Figure 2I, the distance was precisely controlled using a widefield microscope with a 10 × objective, which was incremented from 0 to 400 μm (as shown in Supplementary Figure 1). The CSFOE tip and the hydrophone tip were both immersed in a drop of degassed water placed on a glass microscope slide. The CSFOE was connected to a Q-switched 1,030-nm nanosecond laser (Bright Solution, Inc. Calgary Alberta, CA) with a laser pulse energy of 24, 46, 56, and 65 μJ, respectively. The setup of the measurement is shown in Figure 2D. The acoustic pressure values were calculated based on the calibration curve obtained from the hydrophone manufacturer. The frequency data were obtained through the Fast Fourier Transform (FFT) using *MATLAB R2020a*. For visualizing the acoustic wavefront, a Q-switch Nd: YAG laser (Quantel Laser CFR ICE450) was used to deliver 8 ns pulses to the CSFOE. The generated acoustic signal was capture by a 1 × 128 linear transducer array (L22-14, Verasonics Inc.) and processed by an ultrasound imaging system (Vantage128, Verasonics).

### Embryonic neuron culture

Primary cortical neuron cultures were derived from Sprague-Dawley rats. Cortices were dissected from embryonic day 18 (E18) rats of either sex and then digested in papain (0.5 mg/mL in Earle’s balanced salt solution) (Thermo Fisher Scientific Inc., MA). Dissociated cells were washed with and triturated in 10% heat-inactivated fetal bovine serum (FBS, Atlanta Biologicals, GA, USA), 5% heat-inactivated horse serum (HS, Atlanta Biologicals, GA, USA), 2 mM Glutamine-Dulbecco’s Modified Eagle Medium (DMEM, Thermo Fisher Scientific Inc., MA, USA), and cultured in cell culture dishes (100 mm diameter) for 30 min at 37°C to eliminate glial cells and fibroblasts. The supernatant containing neurons was collected and seeded on poly-D- lysine coated cover glass and incubated in a humidified atmosphere containing 5% CO_2_ at 37°C with 10% FBS + 5% HS + 2 mM glutamine DMEM. After 16 h, the medium was replaced with Neurobasal medium (Thermo Fisher Scientific Inc., MA, USA) containing 2% B27 (Thermo Fisher Scientific Inc., MA, USA), 1% N2 (Thermo Fisher Scientific Inc., MA, USA), and 2 mM glutamine (Thermo Fisher Scientific Inc., MA, USA). On day five, cultures were treated with 5 μM FDU (5-fluoro-2’-deox- yuridine, Sigma-Aldrich, MO, USA) to further reduce the number of glial cells. Additionally on day five, the *pAAV.Syn.GCaMP6f.WPRE.SV40* virus (Addgene, MA, USA) was added to the cultures at a final concentration of 1 μL/mL for *GCaMP6f* expression and has cre-independent expression already with no Cre virus involved in the process. Half of the medium was replaced with fresh culture medium every 3–4 days. Cells cultured *in vitro* for 10–13 days were used for CSFOE stimulation experiment.

### *In vitro* neurostimulation

*In vitro* neurostimulation experiments were performed using a Q-switched 1,030-nm nanosecond laser (Bright Solution, Inc. Calgary Alberta, CA). A 3-D micromanipulator (Thorlabs, Inc., NJ, USA) was used to position the CSFOE in the cell culture dish. Calcium fluorescence imaging was performed on a lab-built wide-field fluorescence microscope based on an Olympus IX71 microscope frame with a 20 × air objective (UPLSAPO20X, 0.75NA, Olympus, MA, USA), illuminated by a 470 nm LED (M470L2, Thorlabs, Inc., NJ, USA) and a dichroic mirror (DMLP505R, Thorlabs, Inc., NJ, USA). Image sequences were acquired with a scientific CMOS camera (Zyla 5.5, Andor) at 20 frames per second. Neurons expressing GCaMP6f at DIV (day *in vitro*) 10–13 were used for the stimulation experiment. For the tetrodotoxin (TTX) control group, tetrodotoxin citrate (ab120055, Abcam, MA, USA) was added to the culture to reach 3 μM final concentration 10 min before Calcium imaging. The fluorescence intensities, data analysis, and exponential curve fitting were analyzed using *ImageJ (Fiji)* and *MATLAB R2020a*.

### Data analysis

Calcium images were analyzed using ImageJ. The fluorescence intensity was measured by selecting the soma. Calcium traces, acoustic waveform and temperature traces were analyzed using *MATLAB R2020a* and *Prism 9*. All statistical analysis was done using *MATLAB R2020a*. All statistical analysis was done using two-sample *t*-test otherwise specified. Data shown are mean ± standard deviation.

## Results

### Simulation of acoustic waveforms generated from candle soot-based fiber optoacoustic emitters

To identify the optimal condition toward maximized optoacoustic conversion efficiency, we used *COMSOL Multiphysics 5.4* to simulate the generation and propagation of the optoacoustic signals. Taking advantage of COMSOL’s ability of multiple physics field simulations, we simulated the different steps of optoacoustic generation: laser absorption, thermal expansion, and acoustic wave propagation. Since the candle soot has a very porous structure, the PDMS diffuses into the candle soot layer, forming a uniformly mixed candle soot/PDMS mixture layer (Chang et al., 2015). Therefore, we included a candle soot/PDMS mixture layer and a pure PDMS layer in the 2D axisymmetric CSFOE model built in *COMSOL Multiphysics 5.4* for simulation (Figure 1B). A single 3 ns laser pulse was delivered through a multimode fiber (with a 200 μm core diameter) to the double layer coating on the fiber tip. Figure 1C shows a representative wave front of the generated ultrasound 400 ns after the onset of the laser, indicating a bipolar pressure signal generated by CSFOE (Figure 1C, red: positive pressure; blue: negative pressure). Figure 1D plots the time domain waveforms when the thickness of the CS/PDMS layer varied from 1 μm to 40 μm. In Figure 1E, the normalized peak-to-peak amplitude of the generated PA signal is plotted as a function of the thickness of the CS/PDMS mixture layer. The optimal thickness of the CS/PDMS layer, which generates the largest amplitude PA signal, was found to be ∼10 μm. This result is consistent with the previous work, where an optimal thickness generating the maximum pressure was also found (Chang et al., 2018).

### Fabrication and characterization of candle soot-based fiber optoacoustic emitters

To fabricate the optimal CSFOE, as guided by the simulations, we developed a two-step fabrication procedure to precisely control the CS layer thickness (Figure 2A). A polished multimode fiber with a 200 μm core diameter (Thorlabs) was inserted into a fiber ferrule. The fiber tip was positioned so that it was flush with the distal end of the ferrule. Then, the distal tips of both the ferrule and fiber were placed into the flame core of a paraffin wax candle, where they were fully coated with flame synthesized candle soot (Figure 2A, left). The key parameter to control the thickness of the CS was the coating time, which ranged between 1 and 20 s. Then, a nanoinjector was used to deposit a controlled amount of PDMS (∼0.01 μm^3^) onto the tip of the fiber coated with candle soot (Figure 2A, middle). The transmission images of the CS-coated fiber before and after PDMS coating are also shown in Figure 2A (right). When varying the CS coating time, the thickness of the CS coating was measured from the transmission images of samples before PDMS coating. Figure 2B plots the thickness of the CS layer measured as a function of deposition time. The CS layer thickness was linearly proportional to the deposition time, with an estimated deposition rate of 3.04 μm/s, similar to previous reports (Chang et al., 2018). Such a linear relation enables us to precisely control the thickness of the CS layer to study the PA conversion as a function of the layer thickness. Transmission of CS layers with different thicknesses were also measured (Figure 2C). The normalized transmission of the coating exponentially decreased as a function of the thickness. An absorption depth, the thickness when the transmission decreased to 1/e of initial transmission at the zero thickness was obtained as 6.6 μm. This measured ultrathin absorption depth indicated strong absorption of CS in NIR, enabling efficient ultrasound generation.

The characterization of the CSFOE with various CS/PDMS layer thicknesses was performed with a 40 μm needle hydrophone. A 1,030 nm nanosecond pulsed laser, with a 46 μJ pulse energy was delivered to the CSFOE to generate optoacoustic signals. The acoustic signals were measured for CSFOEs where the thickness of the CS-PDMS mixture layer ranged from 1 μm to 57 μm (Figure 2D,E). The peak-to-peak pressure is plotted as a function of the CS layer thickness in Figure 2F. An optimal thickness of ∼10 μm was found to generate the highest peak-to-peak pressure of 9 MPa. Notably, the experimentally measured optimal thickness and the trend between the thickness and the peak-to-peak pressure are consistent with the simulation results. Importantly, the 10 μm optimal thickness was also found to be close to the 6.6 μm absorption depth of CS-PDMS layer obtained from the absorption shown in Figure 2C. The greatest optoacoustic conversion efficiency occurred when the absorption layer thickness equaled the material absorption depth. In the thickness range < 10 μm, when increasing the absorption layer thickness, the thickness at the absorption depth allowed complete optical absorption. Further increasing the thickness beyond the absorption depth (> 10 μm) led to acoustic attenuation, as demonstrated in previous works (Chang et al., 2018).

Frequency characterization of the generated optoacoustic signal is shown in Figure 2G. The frequency spectrum of the measured acoustic waveforms after Fast Fourier Transform (FFT) exhibited a peak acoustic frequency of 12.8 MHz. This frequency was similar to previous studies on candle-soot-based optoacoustic films (Chang et al., 2018), in which a central frequency of ∼10 MHz was detected for ∼2 μm CS coating thickness. Based on the frequency analysis of ultrasound signal generated, CSFOE emitted broadband high-frequency ultrasound centered at 12.8 MHz. Previous studies have reported that, for laser-induced ultrasound, the generated ultrasound waveform is mainly determined by the absorption of the material and the laser duration (Lee et al., 2018). For CSFOE, in principle, the central frequency can be controlled by the absorption ability of candle soot through changing the candle soot thickness when the physical thickness is smaller than the absorption depth, which is approximately 6.6 μm. Specifically, thinner thicknesses lead to a higher frequency. It is evident that CSFOE with 1 μm CS produces a central frequency of 27.3 MHz while CSFOE with 12 μm CS has a central frequency of 10.6 MHz (Supplementary Figure 2).

To map the propagation of the optoacoustic wave generated by the CSFOE, the pressure was measured at different distances away from the CSFOE using a 40 μm needle hydrophone as shown in Figure 2H (*N* = 3 for each distance). The peak-to-peak pressure of the generated ultrasound is plotted as a function of distance in Figure 2I. The measurements were repeated for three times and the average values were plotted. The confinement of the generated acoustic field, defined by the distance where the pressure decreases to 1/e of the initial pressure at 0 μm, was found to be ∼300 μm, approximately equal to the size of the fiber core. Such decay of optoacoustic pressure over the distance away from the CSFOE tip enables a sub-millimeter localized neuron stimulation. In addition, Figure 2I shows that the dependence on distance is different from the previous 1/r^2^ relation obtained in FOC. The difference is due to the fact that the ultrasound field emitted by CSFOE is at a higher frequency, therefore propagates more directionally, compared with more omnidirectional propagation of the lower frequency FOCs (Jiang et al., 2020).

The propagation of generated ultrasound can be directly visualized using an optoacoustic tomography system (Figure 2J). The acoustic signal was detected by a 1 × 128 linear transducer array (L22-14, Verasonics Inc.) and processed by an ultrasound imaging system (Vantage128, Verasonics). The emitted ultrasound waveform (red) obtained with a time interval of 0.5 μs and the image of the tip of the CSFOE (yellow) are overlaid in Figure 2J. Through the photoacoustic waveform shown in Figure 2J, the emission angle of CSFOE was measured to be 25.3 degrees. For FOC reported previously (Jiang et al., 2020), the emission angle was measured to be 55.1 degrees which is around twice as large. This observation also supports the more directional propagation for the CSFOE generated ultrasound field (Jiang et al., 2020).

Different laser energy inputs also resulted in varied output pressures. Using different fiber attenuators to control the laser energy input, the waveform of generated acoustic signal was measured by the needle hydrophone (Figure 2K) (*N* = 3 for each energy condition), and the peak-to-peak pressure is plotted as a function of input energy in Figure 2L, showing a fitting curve of *P* = 0.226 * *E* (*R*^2^ = 0.93, fitting coefficient of determination) confirming the linear dependence of the pressure on the input laser energy. We observed potential coating damage when the laser energy was greater than 65 μJ under the pulse train composed of five laser pulses, which limits the maximum pressure the device can produce. We conclude the 15 MPa is the CSFOE pressure up limit under a 1.7 kHz repetition rate, 3 ms pulse train duration (5 pulses) and pulse energy of 56 μJ. It is expected to produce higher pressure driven by energy larger than 56 μJ and a shorter pulse train than five pulses. Through controlling the distance away from the CSFOE tip and laser energy, we can have a complete control of the generated pressure in a large range under 15 MPa for various applications. By rationale fabrication of the layered structure of CSFOE and control of PA pressure generated, CSFOE can serve as a robust device for repeatable neuromodulation and allows us to study neuron responses under different conditions.

### Candle soot-based fiber optoacoustic emitter stimulation of neurons *in vitro*

To confirm the stimulation function of the CSFOE, GCaMP6f-labeled primary neurons (DIV 10-13) were cultured on a glass bottom dish, and calcium imaging was performed to monitor neuronal activities. A 3 ns pulsed laser at 1030 nm with a repetition rate of 1.7 kHz was delivered to the CSFOE. The laser pulse train, with a duration of 3 ms (corresponding to 5 pulses) and pulse energy of 65 μJ, was used for CSFOE optoacoustic in vitro neural stimulation. The CSFOE was precisely controlled by a 4D micromanipulator to approach the target neurons. The distance between the neurons and the CSFOE tip was monitored to make sure neurons were within the sub-millimeter confinement area.

**FIGURE 3:**
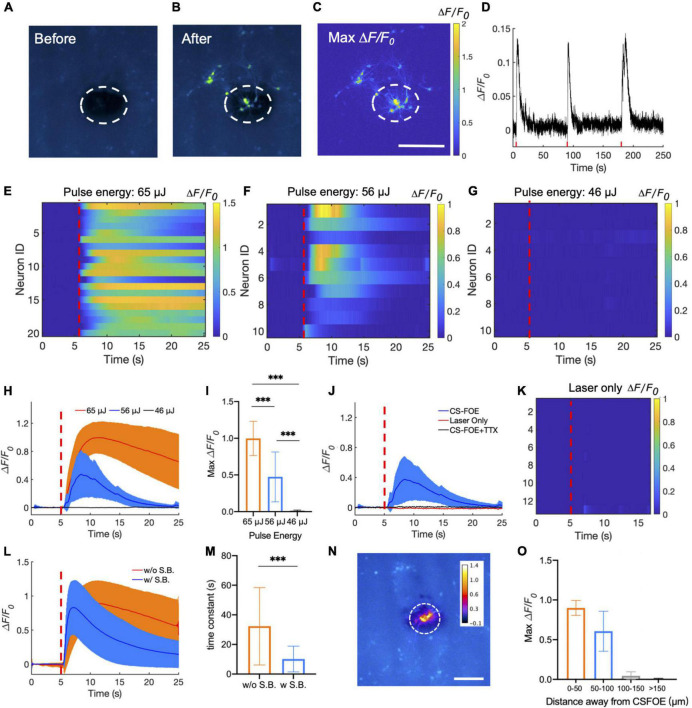
Activation of *GCaMP6f*-expressing cortical neurons by CSFOE stimulation. **(A,B)** Representative fluorescence of neurons from three batches stimulated by CSFOE before stimulation **(A)** and after stimulation **(B)**. **(C)** Map of the maximum fluorescence change Δ*F*/*F*_0_ induced by the CSFOE stimulation. Laser condition: 3 ms duration, pulse energy 65μJ. Scale bar: 200 μm. **(D)** Calcium trace shows repeatable activation of the same neuron. Laser condition: 3 ms duration, pulse energy 56 μJ. **(E–G)** Colormaps of fluorescence change in neurons stimulated by CSFOE with a laser pulse energy of 65 μJ **(E)** (*N* = 20), 56 μJ **(F)** (*N* = 10), and 46 μJ **(G)** (*N* = 10). **(H)** Average calcium traces of neurons obtained from **(E,F,G)** with the pulse energy of 65μJ (Red) (*N* = 20), 56 μJ(*Blue*)(N = 10) and 46 μJ (black) (*N* = 10), respectively. The shaded region corresponds to one standard deviation. Laser turns on at *t* = 5 s (red dashed lines). The duration of each stimulation was fixed at 3 ms. **(I)** Average of maximum fluorescence intensity changes shown in **(E–G)**. Error bars represent standard deviation. ***p < 0.001, one way AVOVA test. **(J)** Average calcium traces of neurons of CSFOE stimulation, Laser only control and TTX control. Each group was repeated three times on three different dishes of neurons (*N* = 10 for each condition). **(K)** Colormaps of fluorescence change in neurons of a laser only control group. **(L)** Average calcium traces with (Red) (*N* = 30) and without (Blue) synaptic blocker (*N* = 30). Laser is on at *t* = 5 s. laser condition: 1,030 nm, 1.7 kHz repetition rate, 65 μJ pulse energy **(M)** Time constant of the decay of the fluorescence trace with and without synaptic blockers. ***p < 0.001, two sample *t*-test. **(N)** Spatial distribution of maximum neuronal calcium response induced by CSFOE stimulation. Scale bar: 200 μm. Dashed circle: indication of the CSFOE position. **(O)** Maximum Δ*F*/*F*_0_ changes over the distance away from the CSFOE (*N* = 6 for 0–50 μm group, *N* = 4 for 50–100 μm group, *N* = 5 for 100–150 μm group, *N* = 7 for > 150 μm group).

Representative fluorescence images of the neuron before and after stimulation are shown in Figures 3A,B. Maximum change of the fluorescence intensity is highlighted in Figure 3C. The dashed circles indicate the location of the CSFOE. Increase in fluorescence intensity Δ*F*/*F*_0_> 10% upon stimulation confirms the successful activation. This map of fluorescence changes in Figure 3C also indicates that neurons within the stimulation area were successfully activated. The activation outside the stimulation area is due to networking effect (more details discussed later). To further investigate whether the CSFOE can activate neurons reliably and repeatedly, we stimulated the same area of neuron three times in 4 min (Figure 3D). Repeatable stimulations were successfully observed after the laser onset at *t* = 5 s, 90 s and 180 s, and all show Δ*F*/*F*_0_ >10%. This result clearly shows that there is no damage caused by CSFOE after stimulation and demonstrates the repeatability and safety of CSFOE stimulation.

Next, we investigated the effect of laser pulse energy on CSFOE stimulation. Each pulse train was fixed to be 3 ms long. Three laser pulse energies, 65, 56, and 46 μJ, were applied to the CSFOE to modulate neural activities. Responses from neurons at each pulse energy are plotted as heatmaps in Figures 3E–G. Representative calcium traces are plotted in Figure 3H. The averages of maximum fluorescence change obtained from these three groups are compared in Figure 3I. With the laser pulse energy of 65 and 56 μJ, neurons showed an average maximum fluorescence change (Δ*F*/*F*_0_) of 99.8 ± 23.3% and 47.4 ± 33.9%, while with laser energy of 46 μJ, the induced fluorescence change is negligible (1.2 ± 1.0%). These results indicate that at the laser pulse train with the repetition rate of 1.7 kHz and 3 ms duration, the activation threshold is between 46 and 56 μJ, corresponding to a pressure of ∼8 MPa. To better statistically analyze the data from different groups, *one-way ANOVA* was used to determine the significant difference. The *P-*value is 5.47 × 10^−7^ between the 65 and 56 μJ groups, 9.56 × 10^−10^ between the 65 and 46 μJ groups, and 1.29 × 10^−5^ between the 56 and 46 μJ (*N* = 20 for 65 μJ group, *N* = 10 for 56 μJ group and *N* = 10 for 46 μJ group), and all groups show significant difference to each other.

To confirm that the observed activation was due to optoacoustic stimulation, we performed a laser-only control and compared it to the calcium traces of CSFOE-stimulated neurons. The laser-only control group used the same optical fiber without any coatings on the tip, and with the same repetition rate of 1.7 kHz, 3 ms duration and laser pulse energy of 56 μJ. No significant fluorescence response was observed in the laser only group (Figures 3J,K). Optical excitation alone triggered negligible activities. Additionally, to investigate whether the activations observed were caused by action potential, we performed a control experiment with addition of 3 mM tetrodotoxin (TTX), a blocker of voltage-gated sodium channels. No significant fluorescence response was observed in the TTX group (Figure 3J), indicating that the observed calcium transients under CSFOE stimulation were induced by the firing of action potentials (*N* = 10 for each condition). These results are also consistent with previous studies of optoacoustic stimulation (Jiang et al., 2021).

To investigate how synaptic inputs affects the stimulation outcomes, we applied a cocktail of synaptic blockers (10 μM NBQX, 10 μM gabazine, and 50 μM DL-AP5). Averaged traces of stimulated neurons with and without synaptic blockers in both conditions are plotted in Figure 3L. Two types of neuron responses were observed: a transient response under synaptic blocking (blue) and a prolonged response without synaptic blocking (orange). The decay portion of the response curves was fitted exponentially. A time constant for the decay was obtained from the time when the fluorescence intensity decreased by a factor of 1/e from the peak fluorescence intensity. The time constant decreased significantly from 32.32 ± 26.15 s without synaptic blocking to 10.15 ± 8.70 s with synaptic blocking. The two group shows significant difference through *t-*test (*p* = 2.75e-04). These results demonstrate that transient stimulation is likely to be the result of direct CSFOE optoacoustic stimulation, while the network effect through synaptic transmission results in prolonged stimulations (Jiang et al., 2021).

To demonstrate the stimulation resolution of CSFOE *in vitro*, we divided all the neurons in the FOV into four parts according to their distance away from the center of the stimulation: 0–50 μm, 50–100 μm, 100–150 μm, 150 μm and above. The averaged maximum Δ*F/F_0_* with standard deviation for neurons in different parts are plotted in Figures 3N,O as follows (*N* = 6 for 0–50 μm group, *N* = 4 for 50–100 μm group, *N* = 5 for 100–150 μm group, *N* = 7 for > 150 μm group). As is shown here, the fluorescence changes of neurons decreased a lot when the distance increased, and no obvious stimulation was observed when the distance is larger than 100 μm. It shows that our CSFOE’s effective stimulation range is highly localized and the effective range is in a circle of a diameter of 200 μm.

### Comparison between candle soot-based fiber optoacoustic emitters and fiber-optoacoustic converters

**FIGURE 4:**
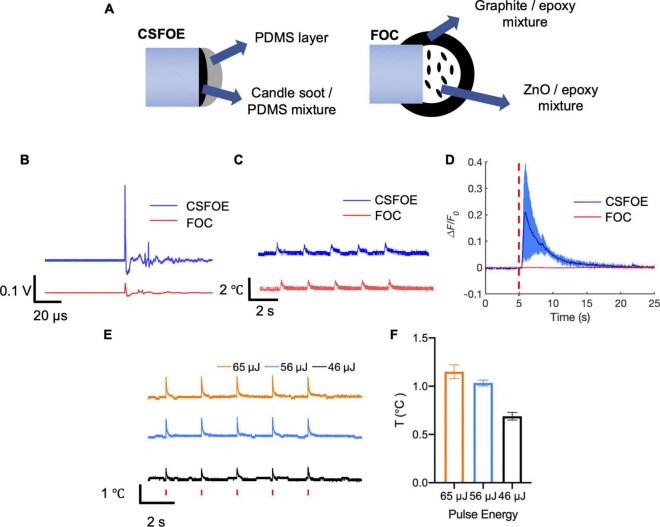
Comparison of CSFOE and FOC. **(A)** Schematics of the CSFOE and FOC. **(B)** Photoacoustic signal of CSFOE and FOC, measured by a 5 MHz transducer under the same laser condition: 1,030 nm, 3 ns, 1.7 kHz, 48 mW. **(C)** Temperature rise measured by a thermal probe placed at the surface of CSFOE and FOC, respectively. **(D)** Representative calcium traces of *GCaMP6f* transfected neurons stimulated by CSFOE (Blue) and FOC (Red) under the same laser energy input of 56 μJ. **(E)** Temperature rise measured by the thermal probe placed at ∼10 μm away from CSFOE under the laser energy used in neuromodulation experiments. Red vertical bars indicate the laser on. Laser condition: 1,030 nm, 1.7 kHz repetition rate, 3 ms duration for each burst. Laser pulse energy is shown in the figure. **(F)** Measured maximum temperature increases at corresponding pulse energy inputs (*N* = 5 for each energy).

To evaluate the performance improvement of CSFOE from the previous FOC fabricated using graphite and epoxy, we first compared the design of CSFOE and FOC. As shown in Figure 4A, both CSFOE and FOC have two-layer structures. Compared with FOC, several improvements were made on the CSFOE regarding the choice of material and structure design. Instead of using a graphite-epoxy system, a CS/PDMS mixture was used in CSFOE as the optoacoustic material. Compared to the previous design, CS has stronger absorption while PDMS is well known for its huge expansion coefficient of 310 μm m^–1^ °C^–1^. The thickness of the CS layer in the CSFOE was optimized to obtain the largest pressure.

To directly compare the performance, we compared the pressure generated by CSFOE and FOC under the same laser condition. A transducer with greater sensitivity compared with the hydrophone was used to measure the generated pressure. As shown in Figure 4B, under the same laser condition of 1,030 nm, 3 ns, 1.7 kHz, 48 mW, CSFOE generated a 9.6 times higher signal than that generated by FOC. In addition, the temperature rise associated with the optoacoustic conversion was measured for both fiber emitters using a thermal coupler (DATAQ DI-245, USA) placed on the surface the fiber tips. According to Figure 4C, the average temperature increases were 0.79°C for the CSFOE and 0.77°C for the FOC. Similar temperature increases suggest that while the CSFOE significantly increased the output pressure, the thermal effect remained minimal. To further study the temperature rise under the condition of *in vitro* experiments, we used the same laser condition and placed CSFOE ∼10 μm away from the thermal probe. As is shown in Figures 4E,F, the near field thermal effect (∼10 μm away from the tip of the CSFOE) was measured by a thermal coupler. The laser condition was kept to be the same, specifically the pulse energies were 46, 56, and 65 μJ (with a 1.7 kHz repetition rate and 3 ms burst duration), respectively. The temperature was measured at approximately 10 μm away from the CSFOE in the cell medium by placing the thermal probe under a microscope. The measured temperature traces were shown in Figure 4E and the maximum temperature increase was shown in Figure 4F. The maximum temperature increases under 46, 56, and 65 μJ were found to be 0.69 ± 0.04, 1.03 ± 0.03, and 1.15 ± 0.07°C respectively, all of which are less than 1.5°C and far below the threshold for photothermal neuron stimulation in which temperature rise exceeded 15°C (Zhu et al., 2022). Such a small temperature increase also minimizes the risk of thermal damage for the neural system.

To compare their performance in neuron modulation, CSFOE and FOC were tested in GCaMP6f labeled neuron culture. Under the laser condition of 3 ms pulse train, 56 μJ pulse energy, 1,030 nm, 1.7 kHz repetition rate, successful activation was observed when CSFOE was applied to neurons. The average maximum Δ*F*/*F*_0_ reached over 20%. When FOC was applied under the same laser condition, no obvious activation occurred (Figure 4D). Notably, previous work showed Ca^2+^ imaging signals indicating successful activation by FOC has been confirmed in Oregon Green labeled neuron culture. GCaMP6f and Oregon Green, as calcium sensors, have different sensitivity upon stimulation. It has been reported that for a single action potential, Oregon Green can generate ∼50% fluorescence change, while GCaMP6f can only generate ∼10% (Palmer et al., 2014; Dana et al., 2019).

To evaluate the average ultrasound intensity during the stimulation, the I_*SPTA*_ and I_*SPPA*_ of the typical CSFOE stimulation of GCaMP6f labeled neurons and FOC stimulation of Oregon Green labeled neurons were calculated according to the equations below.


$$\;I_{SPTA}=\frac{E_{a}}{A\times T}=\frac{A\times \frac{1}{\rho c}\int P{\left(t\right)}^{2}dt}{A\times T}=\frac{\int P{\left(t\right)}^{2}dt}{T\rho c}$$

$$I_{SPPA}=\frac{I_{SPTA}}{DC}$$

Here *E_a_* is the acoustic energy, *A* is the cross section area, *T* is the time for one period (inverse of the repetition rate), ρ is the density of the medium (1,000 kg/m^3^ used for water), *c* is the propagation speed of sound (1,480 m/s in water) and *DC* is the duty cycle of the signal. The repetition rate used in the calculation is 1.7 kHz. Peak-to-peak pressure used for FOC is 0.48 MPa and is 12.4 MPa for CSFOE. CSFOE stimulation has an I_*SPTA*_ of 0.13 W/cm^2^, approximately fifty times larger than the I_*SPTA*_ of 2.46 × 10^–3^ W/cm^2^ for FOC stimulation. Besides, CSFOE has an I_*SPPA*_ of 388.8 W/cm^2^, and for FOC, its I_*SPPA*_ of 0.725 W/cm^2^. Through the calculation above, both CSFOE and FOC are within the safety limit of ultrasound therapy (I_*SPTA*_ < 720 mW/cm^2^) (Pasquinelli et al., 2019), which means there will be no significant tissue damage during the stimulation.

We attribute the difference in the I_*SPTA*_ to the difference in the device frequencies and recording conditions upon stimulation. Specifically, as previously reported the central frequency of FOC is in low-frequency range (∼ 1.5 MHz), while CSFOE generates high frequency ultrasound (12.8 MHz central frequency). The significant difference in ultrasound frequency could lead to different ultrasound intensity required to activate neurons. In addition, in FOC stimulation, Oregon GreenTM 488 BAPTA-1 dextran was used as the calcium sensors, while in CSFOE stimulation we used GCaMP6f. Oregon Green is more sensitive than GCaMP6f in reporting the activation. This difference in sensitivity could lead to different stimulation intensities required for optoacoustic neural stimulation. In short, the different intensity used for stimulation observed in the FOC work and this work could be contributed to different calcium sensors used and the different frequencies of the emitters, collectively.

Collectively, our result clearly shows that CSFOE has a significantly higher stimulation efficacy and can be more widely used for recording based on different kinds of calcium sensors.

### Dual-site neuron stimulation by candle soot-based fiber optoacoustic emitter

**FIGURE 5:**
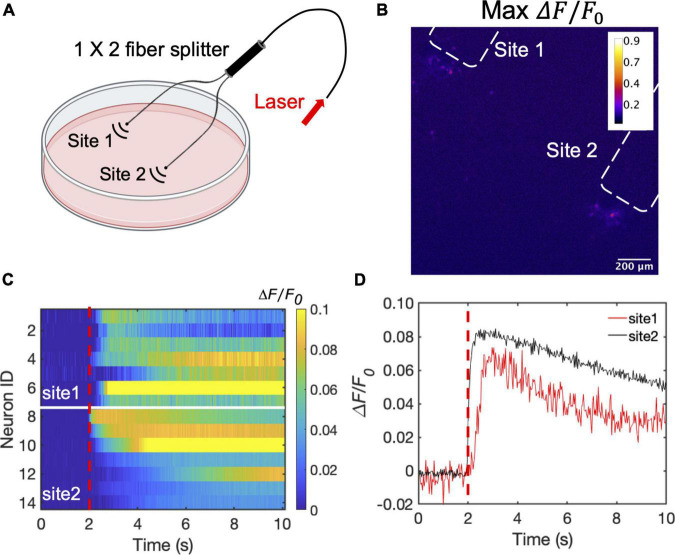
Dual site neuron stimulation by CSFOE. **(A)** Schematic of dual site stimulation using two CSFOEs with a fiber splitter. Created with BioRender.com. **(B)** Map of the max △*F*/*F*_0_ image of two sites of neurons stimulated by two CSFOE. **(C)** Colormaps of fluorescence changes in neurons at two sites stimulated by CSFOE. **(D)** Representative calcium traces from traces shown in **(C)** from neurons at site 1 (Red) and site 2 (Black).

To illustrate the advantage of the high optoacoustic conversion efficiency of CSFOE, we used CSFOE for dual-site neuron stimulation *in vitro*. A 1 × 2 fiber splitter was used for splitting the laser energy into two identical paths. The laser pulse energy of each path was measured to be 53 μJ (90 mW for each site, 1.7 kHz repetition rate) (Figure 5A). As shown in Figure 5B, the map of maximum fluorescence changes MaxΔ*F*/*F*_0_ clearly shows two groups of neurons, with each centered around a CSFOE, being successfully activated by two CSFOEs with fluorescence increase of around 10% at each site. Each group is confined within an area of ∼200 μm associated with the corresponding CSFOE. The highly localized feature of CSFOE stimulation makes it possible to distinguish different sites of stimulation under the same field of view.

Ca^2+^ traces from two groups at these two sites are plotted in a heatmap shown in Figure 5C. Representative traces of 14 cells from different sites are plotted in Figure 5D. Neurons in both sites showed significant change in fluorescence after the laser onset. The fluorescence changes at each site all reached over 10%, which shows that both sites are successfully stimulated (Figure 5C). The high optoacoustic conversion efficiency and the highly localized stimulation area open up potentials for multi-site neuron stimulation.

## Conclusion

In this study, we developed a new fiber optoacoustic emitter based on CS for the first time with high optoacoustic conversion efficiency and demonstrated CSFOE neuromodulation with an improved efficacy compared to FOC. Based on these improvements, we demonstrated dual-site neuromodulation through two CSFOE driven by a single laser source.

To obtain the highest optoacoustic pressure, we chose candle soot as the material of the absorber, which is considered as one of the best materials for optoacoustic generation owing to its high optical absorption. In addition, we optimized the layered design of the CSFOE through both simulation and experiment. The optimized CSFOE was able to generate over 15 MPa peak-to-peak pressure. A more detailed comparison of photoacoustic conversion efficiency between CSFOE and other two fiber optoacoustic emitters used in neuromodulation is shown in Table 1.

**TABLE 1:** Optoacoustic conversion efficiency comparison of different fiber based optoacoustic emitters for neuromodulation.

	CSFOE (this work)	TFOE (Shi et al., 2021)	FOC (Jiang et al., 2020)
Energy conversion efficiency (%)	1.5E-3	2.28E-6	3.14E-5
Optoacoustic conversion efficiency in pressure (Pa m^2^/J)	15,600	130	1,374

Through the direct comparison, CSFOE is ∼ 100 times more efficient than TFOE. Besides, CSFOE shows ∼10 times higher conversion efficiency compared with FOC, which is also evident in the results shown in Figure 4B.

Detailed optoacoustic characterization for CSFOE has also been performed, including power-pressure dependence and distance-pressure dependence. The output optoacoustic peak to peak pressure is linearly proportional to the input pulse energy as *P* = 0.226 * *E*. The distance-pressure dependence confirmed a highly localized ultrasound field of around 200 μm. Based on the results of optoacoustic characterization, we can precisely control the ultrasound intensity to be delivered to neurons by controlling the energy of the laser as well as the distance between CSFOE and neurons.

Successful CSFOE neuron activation has been demonstrated using Calcium imaging. It was found that under the pulse energy of 56 and 65 μJ, at the repetition rate of 1.7 kHz, over a 3 ms duration, the maximum fluorescence change of the stimulated neurons were 47.4 ± 33.9% to 99.8 ± 23.3%, respectively. These laser conditions correspond to optoacoustic pressure of 8.8 MPa and 12.4 MPa at the peak of frequency of 12.8 MHz for CSFOE.

Taking advantage of its high energy conversion efficiency, we performed the dual-site neuron stimulation using two CSFOEs driven by a single laser, which is not feasible by previous fiber based optoacoustic emitters. Dual-site stimulation has lots of potential applications in animal behavior studies, since complex animal behavior is normally controlled by multiple functional area in the brain. CSFOE, offering a superior sub-millimeter spatial resolution and high-pressure conversion efficiency, has the potential to modulate more complex animal behavior by controlling multiple target sites in the circuitry.

In summary, this robust and highly efficient optoacoustic converter, with an easy and repeatable fabrication process, offers a new tool for effective neuron stimulation. With an improved efficiency and the ability to perform multi-site stimulation, CSFOE opens up a great potential for complex animal behaviors that needs multiple stimuli at different locations in a programmable manner.

## Data availability statement

The raw data supporting the conclusions of this article will be made available by the authors, upon requests.

## Ethics statement

This animal study was reviewed and approved by the Institutional Animal Care and Use Committee of Boston University.

## Author contributions

GC, LS, J-XC, and CY: drafting and refining the manuscript. GC: conducting of the simulation. GC and LS: conducting of the experiments. LL, J-XC, and CY: critical guidance of the project. RW, YL, ZD, and MH: help with experiments. LL, YL, and MH: critical reading of the manuscript. All authors have read and approved the manuscript.
