# Supplementary material for: High-precision neural stimulation by a highly efficient candle soot fiber optoacoustic emitter
Source: Front Neurosci. 2022 Nov 3;16:1005810. doi: 10.3389/fnins.2022.1005810 (PMC9669258; doi:10.3389/fnins.2022.1005810)
Supplement: Supplementary file 1 [file Image_1.pdf]

Supplementary Material

1. Supplementary Figures

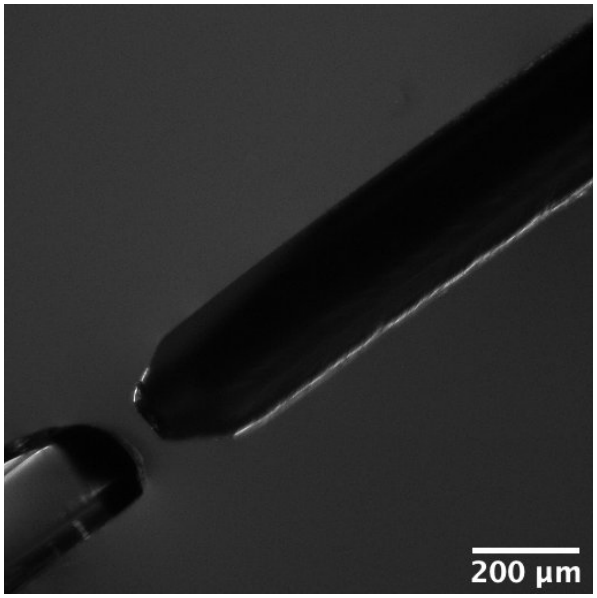

Supplementary Figure 1. Image of CSFOE (lower left) photoacoustic signal measurement using a needle hydrophone (upper right) by a microscope with 10X objective.

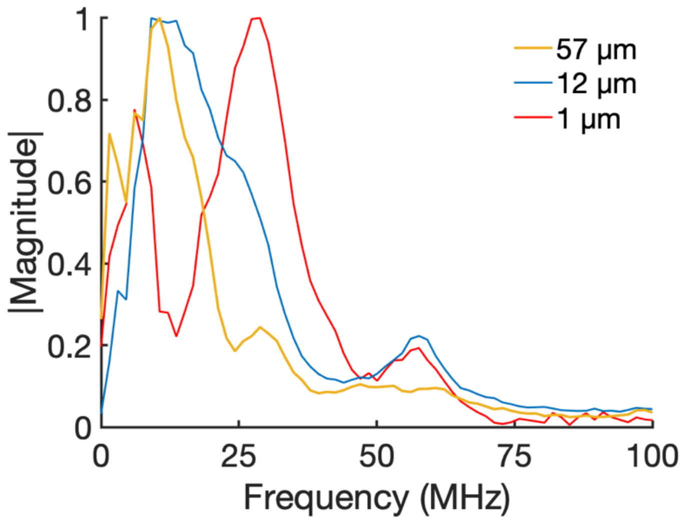

Supplementary Figure 2. Frequency analysis of optoacoustic signal generated by different CSFOEs with different candle soot layer thickness.
